# eHealth Apps Replacing or Complementing Health Care Contacts: Scoping Review on Adverse Effects

**DOI:** 10.2196/10736

**Published:** 2019-03-01

**Authors:** Wilhelmina Josepha Maria Stevens, Rob van der Sande, Lilian J Beijer, Maarten GM Gerritsen, Willem JJ Assendelft

**Affiliations:** 1 Faculty of Health Hogeschool van Arnhem en Nijmegen University of Applied Sciences Nijmegen Netherlands; 2 Department for Primary and Community Care Radboud University Medical Centre Nijmegen Netherlands

**Keywords:** eHealth, adverse effects, scoping review

## Abstract

**Background:**

The use of eHealth has increased tremendously in recent years. eHealth is generally considered to have a positive effect on health care quality and to be a promising alternative to face-to-face health care contacts. Surprisingly little is known about possible adverse effects of eHealth apps.

**Objective:**

We conducted a scoping review on empirical research into adverse effects of eHealth apps that aim to deliver health care at a distance. We investigated whether adverse effects were reported and the nature and quality of research into these possible adverse effects.

**Methods:**

For this scoping review, we followed the five steps of Arksey and O’Malley’s scoping review methodology. We searched specifically for studies into eHealth apps that replaced or complemented the face-to-face contact between a health professional and a patient in the context of treatment, health monitoring, or supporting self-management. Studies were included when eHealth and adverse effects were mentioned in the title or abstract and when empirical data on adverse effects were provided. All health conditions, with the exception of mental health conditions, all ages, and all sample sizes were included. We examined the literature published between December 2012 and August 2017 in the following databases: PubMed, Cumulative Index to Nursing and Allied Health Literature (CINAHL), Web of Science, and the Cochrane Library. The methodological quality of the studies was assessed using the Critical Appraisal Skills Programme (CASP) checklists.

**Results:**

Our search identified 79 papers that were potentially relevant; 11 studies met our inclusion criteria after screening. These studies differed in many ways and the majority were characterized by small research populations and low study quality. Adverse effects are rarely subject to systematic scientific research. So far, information on real adverse effects is mainly limited to incidental reporting or as a bycatch from qualitative pilot studies. Despite the shortage of solid research, we found some indications of possible negative impact on patient-centeredness and efficiency, such as less transparency in the relationship between health professionals and patients and time-consuming work routines.

**Conclusions:**

There is a lack of high-quality empirical research on adverse effects of eHealth apps that replace or complement face-to-face care. While the development of eHealth apps is ongoing, the knowledge with regard to possible adverse effects is limited. The available research often focuses on efficacy, added value, implementation issues, use, and satisfaction, whereas adverse effects are underexplored. A better understanding of possible adverse effects could be a starting point in improving the positive impact of eHealth-based health care delivery.

## Introduction

The use of eHealth has increased considerably in recent years. eHealth comprises all kinds of information and communication technologies, such as websites and apps for screening, assessment and self-monitoring, health promotion, physical training, and social support (eg, video-chat sessions with a therapist, moderated bulletin boards, chat rooms, and social media) [1,2]. eHealth is generally considered to have a positive effect on health care quality and to be a promising alternative to face-to-face health care contacts [3]. Moreover, the use of eHealth apps is expected to reduce health care consumption and health care costs [4]. Also, eHealth is supposed to contribute to the fast availability of updated medical information, as well as to the provision of tailored care, independent of place and time [5]. In addition, although research in the field is not conclusive, eHealth may improve self-management, health literacy, and healthy behavior [6,7].

In line with these high expectations, large companies and health care organizations invest many millions of dollars in the development of eHealth apps [8,9]. Given the large investments into eHealth, it is remarkable that there is a lack of information with regard to possible unfavorable outcomes of eHealth interventions. It is not known whether there are any adverse effects or whether they are not included in scientific research. The limited number of studies in this field have reported, for example, adverse events such as patients’ anxiety caused by monitoring vital signs [10]. In another publication, it was mentioned that professionals get overwhelmed by the amount of data, workload, and workarounds [11].

We conducted a scoping review on empirical studies into adverse effects of eHealth apps that aim to deliver health care at a distance. We welcome the advantages that eHealth interventions will bring; a better understanding of what is known about possible adverse effects will help to improve the use of eHealth.

## Methods

### Overview of Scoping Review Methodology

For this scoping review, we followed the five steps according to Arksey and O’Malley’s scoping review methodology, supplemented with recommendations by Levac et al and Daudt et al [12-14]. We used this method because the aim of a scoping review is to assess the available research literature in order to chart the nature, range, and extent of the research evidence and to identify gaps in the existing literature [15].

### Step 1: Identifying the Research Question

In our study, we defined eHealth as the use of information and communication technologies to support or improve health care and health care delivery [1,16]. We searched specifically for studies into eHealth apps that replaced or complemented the face-to-face contact between a health professional and a patient in the context of treatment, health monitoring, supporting self-management, or their communication [17]. Examples are online tools for patients with a chronic condition that replace some of the outpatient checks with online care or online rehabilitation programs that integrate outpatient treatment with exercises performed at home. An adverse effect was defined as any unfavorable outcome on the quality of care that occurred as a result of the use of an eHealth intervention [18].

The following research questions were formulated:

1. Which adverse effects of eHealth apps are reported in empirical studies?
2. What is the nature and quality of the research into the adverse effects of eHealth apps?

### Step 2: Identifying Relevant Studies

We examined the literature published between December 2012 and August 2017 in the following databases: PubMed, the Cumulative Index to Nursing and Allied Health Literature (CINAHL), Web of Science, and the Cochrane Library. We used RefWorks 2.0 (ProQuest), a Web-based bibliographic manager, to import all citations. After an initial broad search and consultations with a librarian, the final search query was composed; we used the set of comprehensive Medical Subject Headings (MeSH) and the free-text search term *eHealth* or its synonyms, as well as the term *adverse effects* or its synonyms. To find as many relevant articles as possible, we decided to add *quality of care* and *risks* as title words because both appeared to be related to articles on adverse effects (see Multimedia Appendix 1 for search query).

### Step 3: Selecting Studies

The primary search resulted in 6010 records. After removing duplicates, 5523 titles and abstracts were screened for relevance and for the inclusion and exclusion criteria by two researchers, independently (WJMS and MGMG). Our aim was to include articles that described apps that replaced or complemented the face-to-face contact between a health professional and a patient in the context of treatment, health monitoring, supporting self-management, or their communication. Studies were also included when eHealth and adverse effects were mentioned in the title or abstract and when empirical data—qualitative or quantitative—on adverse effects were provided. Adverse effects had to be related to patients or quality of care.

We wanted a broad search and, at the same time, homogeneity in apps. Mental health conditions were excluded from our scoping study, as the eHealth studies found were already very diverse and this would make the diversity too great. We have tried to achieve as much homogeneity as possible. Except for mental health conditions, all other health conditions, ages, and sample sizes were included. The papers had to be written in English and published between December 2012 and August 2017. We did not use studies in the field of public health or studies on electronic health records, electronic medical records, education, or surgical technology. Exchange of patient information between stakeholders generates different problems and challenges, such as technical matters and privacy issues. We also excluded adverse effects related to security and privacy of data storage and transmission. Prior to inclusion, two of the authors (WJMS and MGMG) verified their agreement in applying the inclusion criteria. Disagreement was solved by discussion. In case no consensus was reached, a third expert (RvdS) was consulted. This resulted in 79 studies meeting the inclusion criteria.

**Figure 1:**
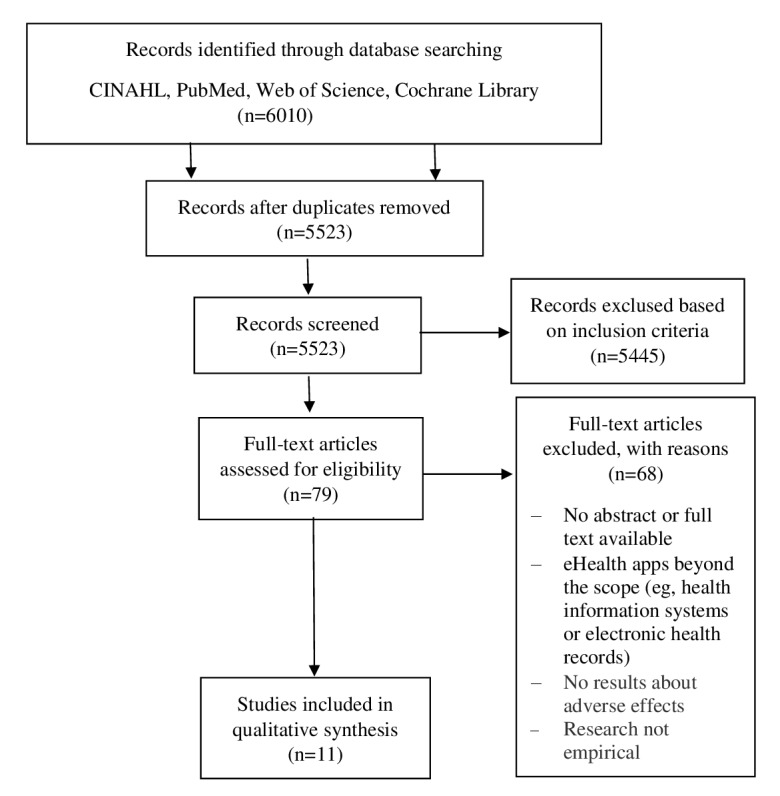
Study selection process. CINAHL: Cumulative Index to Nursing and Allied Health Literature.

Two of the authors (WJMS and MGMG) subsequently screened the full text of the selected articles, independently, for information on adverse events. The screening results were compared and any discrepancies were resolved by discussion. If the outcome was unclear, two other authors (LJB and RvdS) from the research team arbitrated. For the final synthesis, we excluded 68 studies that did not meet the inclusion criteria, leaving 11 studies for final synthesis (see Figure 1). Study quality was independently evaluated by two researchers (WJMS and MGMG) and disagreements were resolved through discussion in order to reach final study-quality ratings.

### Step 4: Charting the Data

We extracted and summarized information for author, year, geographic area, title, name and function of the intervention, study population, study design, outcome and measurements, results, and conclusions of adverse effects. All articles were assessed and data were extracted independently by WJMS and MGMG.

We used the Critical Appraisal Skills Programme (CASP) Qualitative Research Checklist and the Randomised Controlled Trial Checklist for appraisal [19,20]. WJMS and MGMG assessed the studies independently and any disagreement was resolved by discussion. Scores were displayed as the proportion of the number items filled in with *yes* in relation to the total number of items (see Multimedia Appendix 2 for overview).

### Step 5: Collating, Summarizing, and Reporting the Results

In this phase, results related to adverse effects were discussed by the authors WJMS and MGMG and by the experts RvdS, LJB, and WJJA. Results were categorized following the six domains of quality as formulated by the Agency for Healthcare Research and Quality (AHRQ): safety , effectiveness, patient-centeredness, timeliness, efficiency, and equitability [21,22].

## Results

### Overview

We found 11 studies that met our inclusion criteria. The characteristics of included studies are summarized in Multimedia Appendix 3. A total of 9 studies out of 11 (82%) used qualitative data. Observation, semistructured interviews, questionnaires, and documentary study are the forms of qualitative research that we have come across in the selected studies.

### General Characteristics

#### eHealth Terminology and Functions

Different terms were used for the eHealth app within articles: telerehabilitation [23], telecare [24,25], telemonitoring [26], telemedicine consultation [27], video telehealth [28], video teleconsultation [29], Internet intervention [30], eVisit [31], mobile health [32], and digital communication [33].

These various apps served different functions: supporting exercise [23,24,32], (video)consultation [25,27,29,33], supporting self-management [25,26,30], triage [28], and primary care [31].

#### Study Participants

Study participants were patients [23,27,29-32], health care professionals [25], or a mix of patients and health care professionals [24,26,28,33].

A total of 8 out of 11 studies (73%) involved an intervention targeting patients with a chronic health problem [23-26,29,30,32,33].

#### Study Design and Quality

A total of 8 out of 11 studies (73%) had a quantitative design, of which 2 studies (25%) were randomized controlled trials (RCTs). Multimedia Appendix 2 shows an overview of the quality appraisal.

Study quality, in general, was very low. The sample size in the studies varied from 2 [28] to 564 [31] participants. There were 2 studies out of 11 (18%) that explicitly used adverse effects, a priori, as a primary outcome measure.

### Adverse Effects

#### Overview

Table 1 shows study results from studies about adverse effects categorized following the AHRQ six domains of quality.

#### Safety

Griffiths et al performed a study into the use of text messages, email, and social media in the communication between young people and a clinical team. They found an increased risk of communication failures, failure to record the content of the communication, and failure to consult the patient’s notes prior to engaging in communication [33].

Furthermore, in the studies of Benvenutti et al and Buvik et al, safety was part of the outcome. In both studies, the eHealth intervention was judged to be safe because no study-related adverse events were observed among, or reported by, study participants [23,27].

#### Effectiveness

In an RCT by Petrella et al, an online exercise program for people with metabolic risk was used. After 12 weeks, this program resulted in a lower systolic blood pressure (SBP) in the active control group compared to the eHealth group. By 52 weeks, the reduction in SBP was similar in both groups [32].

#### Patient-Centeredness

The study by Bodker et al showed that when eHealth interventions aimed to support patients in doing their physical exercise at home, responsibilities between the health professional and patient became less transparent [24].

A total of 3 out of 11 articles (27%) reported a lack of human face-to-face contact between the health professional and patient or reported on the impossibility for physical examination, leading to “...a new perceptual distance between patients and therapists” [24,29,30].

eHealth apps that support self-management cause patients to express a sense of loss of privacy and stigmatization [25], loss of trust by patients [30], or poor cooperation or lack of willingness on the part of patients [25,30]. Fairbrother et al explored patient and professional views on self-management in the context of telemonitoring. They reported that professionals expressed concerns about promoting the sick role and creating dependence on telemonitoring and professionals [26].

#### Efficiency

In the study by Bodker et al, health care professionals mentioned new time-consuming work routines (ie, a significant amount of coordination tasks) [24]. Cady et al investigated triage nurse workflow before and after the implementation of video telehealth. They found an increased triage time [28]. Buvik et al, however, found no differences in consultation duration in their RCT investigation into video-assisted remote orthopedic consultations in an orthopedic outpatient clinic [27].

In a study by Mehrotra et al on a comparison of care between eVisits and physician office visits, physicians using eHealth were less likely to order relevant tests or order preventive care and researchers established the occurrence of overprescribing of antibiotics [31].

**Table 1:** Reported results from studies about adverse effects.

AHRQ^a^ domains of quality	Adverse effects
Safety	No study-related adverse events [23] No serious adverse advents were found to be related to the mode of the consultation [27] Communication failures [33] Failure to record the content of the communication [33] Failure to consult the patient’s notes prior to engaging in communication [33]
Effectiveness	No difference in re-referrals [27] Reduction in SBP^b^ greater in control group at 12 weeks and similar at 52 weeks [32]
Patient-centeredness	A new perceptual distance between patients and therapists [24] Sense of losing privacy [25] Stigmatization [25] Poor cooperation of the patient [25] Loss of trust [30] Lack of human face-to-face contact [30] Lack of willingness [30] Concerns about promoting the sick role [26] Creates dependence on telemonitoring and professionals [26] Lack of physical contact causes concerns about long-term complications of diabetes [29]
Timeliness	N/A^c^
Efficiency	New time-consuming work routines [24] Responsibilities less transparent [24] Mean consultation duration not different [27] Increased triage time [28] Less likely to order urinary tract infection-relevant tests [31] No difference for follow-up visits [31] Overprescribing of antibiotics [31] Less likely to order preventive care [31]
Equitability	N/A^c^

^a^AHRQ: Agency for Healthcare Research and Quality.

^b^SBP: systolic blood pressure.

^c^N/A: not applicable, as no adverse effects related to timeliness and equitability were reported in the selected studies.

#### Timeliness and Equitability

Adverse effects related to timeliness and equitability were not reported in the selected studies.

## Discussion

### Principal Findings

Our scoping review shows that there is a clear lack of empirical research on adverse effects of eHealth apps that replace or complement face-to-face care. After a broad search for empirical studies, we were only able to include 11 studies. These studies differed not only in the function (eg, monitoring or assessment) of the eHealth intervention, but also in study population, methodology, and outcome; the majority of the studies entailed small research populations and low study quality.

Adverse effects are rarely subject to systematic scientific research. So far, information on real adverse effects is mainly limited to incidental reporting or as a bycatch from qualitative pilot studies. The diversity and the low quality among studies in our scoping review do not provide a good understanding of the nature and size of these possible adverse effects and do not offer a good enough understanding to make a meaningful benefit-harm analysis.

Despite this shortage of solid research, we suggest that eHealth may have a negative impact on the transparency of the relationship between health professionals and patients regarding their responsibilities [24]. Furthermore, because in some apps there is no nonverbal communication and no ability to perform a physical examination, health professionals worry about the effect of eHealth on the quality of the communication that will, in turn, affect the quality of care. Confidentiality issues and potential negative feelings can arise as a result of this changing relationship [28]. Patients may overemphasize the impact of their condition by getting *fixated* on readings and the monitoring of data. This can lead to dependence on telemonitoring and professionals [26,33].

Thereby, complex programs of therapeutic exercises delivered by technology had limited success in engaging people in chronic pain. Patients showed a lack of willingness and engagement because they missed some help and face-to-face acknowledgement or the content did not seem very relevant to them [30].

Furthermore, our findings show that eHealth may have an adverse effect on efficiency because of new time-consuming work routines [24,28]. Bodker et al reported subtle transformations of work activities, such as recruiting patients, conducting home visits to give personalized advice on home training, and invisible work necessary to uphold the telerehabilitation infrastructure.

In the study by Cady et al, time spent on video triage activities was significantly longer than the time spent on equivalent telephone triage. Their workflow analysis revealed that new activities were added, such as preparing for video telehealth sessions, troubleshooting, and the possibility to arrange an appointment with the physician to participate in the session. In addition, the possibility to interact not only with the parent(s), but also with the child during the video telehealth assessment, caused an increased workflow [28].

### Limitations

Various limitations of this scoping review need to be considered. Some articles may have been missed when the search was undertaken. Due to the research question, we only searched for empirical studies that primarily focused on adverse effects. Studies may not be explicit about their findings related to adverse effects in the title or the abstract, as it may not be the main goal of the study. In addition, alternative terminology is used, such as *unintended consequences*, *negative effects,* or *quality of care* to report relevant findings , which means this review may not be inclusive of all papers that have reported relevant results.

Furthermore, we searched specifically for eHealth apps that delivered health care at a distance. It is possible that we missed studies that did not meet the inclusion criteria, but that do offer value in understanding the phenomenon of adverse effects of eHealth. Our findings address different eHealth apps, goals, and implementation contexts; different users, communities, and countries; and different chronic conditions. Due to the small number of often poorly qualitative studies and the diversity of the apps examined, we believe that our findings cannot apply to eHealth in general.

### Comparison With Prior Literature

The concept *eHealth* is a relatively new way of providing care and is used for different applications, technologies, and care processes. Although the number of articles reporting on eHealth interventions has increased in the past 10 years, it is still a relatively new field of research. Most eHealth interventions are now at a pilot phase and as their implementation is often halted by organizational, cultural, or financial barriers, most studies focus on implementation and organizational issues.

In an overview of systematic reviews of studies into the impact of telehealth care on the quality and safety of care in 2013, McLean et al report, “It was not clear whether adverse events did not occur or whether there was a lack of reporting.” They did not come across any studies that explicitly examined impacts of telehealth care on patient safety [34]. Our findings confirm that we still do not know if there are adverse effects or if the issue of adverse effects is simply not addressed.

Research on the risks of eHealth have mostly focused on factors such as infrastructure, technological issues, implementation issues, and lower adherence. Unfavorable patient outcomes are rarely mentioned [11,34,35].

### Recommendations for Future Design and Research

While this scoping review highlights few adverse effects of eHealth interventions, there remains a gap in empirical research that should be addressed in the future. Researchers need to consider and anticipate these adverse effects of eHealth interventions. The changing relationship between, and responsibilities of, health professionals and patients, greater dependence of patients on health care, potential negative feelings, and new time-consuming work routines are important subjects for research in the future. Furthermore, for future research, it is worthwhile to discriminate apps used as replacements or as complements to regular care. A proper insight into the size and nature of adverse effects will only arise if these effects are systematically investigated, preferably by RCTs. We therefore recommend that adverse effects be included as a standard in studies into the effects of eHealth apps.

### Conclusions

eHealth may contribute to more accessible and more efficient health care. So far, possible negative effects have not been thoroughly investigated. The little research that has been done suggests that they do exist. Given the rapid expansion of eHealth, there is an urgent need for further research on this issue.

## Appendix

Multimedia Appendix 1 Search strategies for the different online databases.

Multimedia Appendix 2 Appraisal of studies by study design using Critical Appraisal Skills Programme (CASP) tools.

Multimedia Appendix 3 Description of the 11 included articles.

## Abbreviations

AHRQ: Agency for Healthcare Research and Quality
CASP: Critical Appraisal Skills Programme
CINAHL: Cumulative Index to Nursing and Allied Health Literature
MeSH: Medical Subject Headings
N/A: not applicable
RCT: randomized controlled trial
