# Supplementary material for: eHealth Apps Replacing or Complementing Health Care Contacts: Scoping Review on Adverse Effects
Source: J Med Internet Res. 2019 Mar 1;21(3):e10736. doi: 10.2196/10736 (PMC6421717; doi:10.2196/10736)
Supplement: Multimedia Appendix 1 [file jmir_v21i3e10736_app1.pdf]

Database	Search strategy
**PubMed**<br>1649 hits	(("Patient Harm"[Mesh] OR "Patient Safety"[Mesh] OR patient risk*[tiab] OR adverse effect*[tiab] OR side effect*[tiab] OR undesirable effect*[tiab] OR injurious effect*[tiab] OR patient safety[tiab] OR patient harm[tiab] OR medical error*[tiab] OR unintended[tiab] OR unexpected[tiab] OR threat*[tiab] OR secondary effect*[tiab] OR quality improvement[mesh] OR quality improvement[tiab] OR quality of care[ti] OR quality of health care[ti] OR quality of healthcare[ti]) AND ("Telemedicine"[Mesh] OR telemedicine[tiab] OR Mobile Health[tiab] OR mHealth[tiab] OR telehealth[tiab] OR eHealth[tiab] OR e-health[tiab] OR e-medicine[tiab] OR emedicine[tiab] OR etherapy[tiab] OR e-therapy[tiab] OR web-based application*[tiab] OR Web based app*[tiab] OR web based self help[tiab] OR web based self management[tiab] OR web based self monitoring[tiab] OR web based self report[tiab] OR web based self screening[tiab] OR Web based Intervention*[tiab] OR internet delivered[tiab] OR blended care[tiab] OR telecare[tiab] OR Telerehabilitation*[tiab] OR Virtual Rehabilitation[tiab] OR Virtual Rehabilitations[tiab] OR Tele-rehabilitation[tiab] OR Tele rehabilitation[tiab] OR Tele-rehabilitation*[tiab] OR Remote Rehabilitation[tiab] OR Remote Rehabilitations[tiab] OR Health Information Technology[tiab] OR Health Information Technologies[tiab] OR Digital health[tiab] OR e-consult*[tiab] OR econsult*[tiab] OR electronic consult*[tiab] OR remote health*[tiab] OR remote consult*[tiab] OR remote counsel*[tiab] OR remote monitor*[tiab] OR remote therap*[tiab] OR remote treatment*[tiab] OR medical app[tiab] OR medical apps[tiab] OR health app[tiab] OR health apps[tiab] OR m-health[tiab] OR medical health technology[tiab] OR Medical application*[tiab] OR health application*[tiab])) OR (telemedicine[ti] OR Mobile Health[ti] OR mHealth[ti] OR telehealth[ti] OR eHealth[ti] OR e-health[ti] OR e-medicine[ti] OR emedicine[ti] OR etherapy[ti] OR e-therapy[ti] OR telecare[ti] OR Telerehabilitation*[ti] AND (risk[ti] OR risks[ti]))
**Web of Science**<br>1799 hits	TS=((patient NEAR/1 risk*) OR (adverse NEAR/1 effect*) OR (side NEAR/1 effect*) OR (undesirable NEAR/1 effect*) OR (injurious NEAR/1 effect*) OR (patient NEAR/1 safety) OR (patient NEAR/1 harm) OR (medical NEAR/1 error*) OR unintended OR unexpected OR threat* OR (secondary NEAR/1 effect*) OR (quality NEAR/1 improvement) OR “quality of care” OR “quality of health care” OR “quality of healthcare”) AND TS=(telemedicine OR Mobile NEAR/1 Health OR mHealth OR telehealth OR eHealth OR e-health OR e-medicine OR emedicine OR etherapy OR e-therapy OR (web-based NEAR/1 application*) OR (Web NEAR/1 based NEAR/1 app*) OR (web NEAR/1 based NEAR/1 self NEAR/1 help) OR (web NEAR/1 based NEAR/1 self NEAR/1 management) OR (web NEAR/1 based NEAR/1 self NEAR/1 monitoring) OR (web NEAR/1 based NEAR/1 self NEAR/1 report) OR (web NEAR/1 based NEAR/1 self NEAR/1 screening) OR (Web NEAR/1 based NEAR/1 Intervention*) OR (internet NEAR/1 delivered) OR (blended NEAR/1 care) OR telecare OR Telerehabilitation* OR (Virtual NEAR/1 Rehabilitation) OR (Virtual NEAR/1 Rehabilitations) OR Tele-rehabilitation OR (Tele NEAR/1 rehabilitation) OR Tele-rehabilitation* OR (Remote NEAR/1 Rehabilitation) OR (Remote NEAR/1 Rehabilitations) OR (Health NEAR/1 Information NEAR/1 Technology) OR (Health NEAR/1 Information NEAR/1 Technologies) OR (Digital NEAR/1 health) OR e-consult* OR econsult* OR (electronic NEAR/1 consult*) OR (remote NEAR/1 health*) OR (remote NEAR/1 consult*) OR (remote NEAR/1 counsel*) OR (remote NEAR/1 monitor*) OR (remote NEAR/1 therap*) OR (remote NEAR/1 treatment*) OR (medical NEAR/1 app) OR (medical NEAR/1 apps) OR (health NEAR/1 app) OR (health NEAR/1 apps) OR m-health OR (medical NEAR/1 health NEAR/1 technology) OR (Medical NEAR/1 application*) OR (health NEAR/1 application*) OR ((telemedicine OR Mobile Health OR mHealth OR telehealth OR eHealth OR e-health OR e-medicine OR emedicine OR etherapy OR e-therapy OR telecare OR Telerehabilitation*) AND (risk OR risks)))
**Cochrane**<br>231 hits	(patient NEAR/2 risk*) OR (adverse NEAR/2 effect*) OR (side NEAR/2 effect*) OR (undesirable NEAR/2 effect*) OR (injurious NEAR/2 effect*) OR (patient NEAR/2 safety) OR (patient NEAR/2 harm) OR (medical NEAR/2 error*) OR unintended OR unexpected OR threat* OR (secondary NEAR/2 effect*) OR (quality NEAR/1 improvement):ti,ab,kw“quality of care” OR “quality of health care” OR “quality of healthcare”:TI AND (telemedicine OR Mobile NEAR/2 Health OR mHealth OR telehealth OR eHealth OR e-health OR e-medicine OR emedicine OR etherapy OR e-therapy OR (web-based NEAR/2 application*) OR (Web NEAR/2 based NEAR/2 app*) OR (web

	NEAR/2 based NEAR/2 self NEAR/2 help) OR (web NEAR/2 based NEAR/2 self NEAR/2 management) OR (web NEAR/2 based NEAR/2 self NEAR/2 monitoring) OR (web NEAR/2 based NEAR/2 self NEAR/2 report) OR (web NEAR/2 based NEAR/2 self NEAR/2 screening) OR (Web NEAR/2 based NEAR/2 Intervention*) OR (internet NEAR/2 delivered) OR (blended NEAR/2 care) OR telecare OR Telerehabilitation* OR (Virtual NEAR/2 Rehabilitation) OR (Virtual NEAR/2 Rehabilitations) OR Tele-rehabilitation OR (Tele NEAR/2 rehabilitation) OR Tele-rehabilitation* OR (Remote NEAR/2 Rehabilitation) OR (Remote NEAR/2 Rehabilitations) OR (Health NEAR/2 Information NEAR/2 Technology) OR (Health NEAR/2 Information NEAR/2 Technologies) OR (Digital NEAR/2 health) OR e-consult* OR econsult* OR (electronic NEAR/2 consult*) OR (remote NEAR/2 health*) OR (remote NEAR/2 consult*) OR (remote NEAR/2 counsel*) OR (remote NEAR/2 monitor*) OR (remote NEAR/2 therap*) OR (remote NEAR/2 treatment*) OR (medical NEAR/2 app) OR (medical NEAR/2 apps) OR (health NEAR/2 app) OR (health NEAR/2 apps) OR m-health OR (medical NEAR/2 health NEAR/2 technology) OR (Medical NEAR/2 application*) OR (health NEAR/2 application*) OR ((telemedicine OR Mobile Health OR mHealth OR telehealth OR eHealth OR e-health OR e-medicine OR emedicine OR etherapy OR e-therapy OR telecare OR Telerehabilitation*) AND (risk OR risks))):ti,ab,kw
**CINAHL** 2331 hits	(MH Patient Safety+ OR (TI ((patient N2 risk*) OR (adverse N2 effect*) OR (side N2 effect*) OR (undesirable N2 effect*) OR (injurious N2 effect*) OR (patient N2 safety) OR (patient N2 harm) OR (medical N2 error*) OR unintended OR unexpected OR threat* OR (secondary N2 effect*)) OR (AB ((patient N2 risk*) OR (adverse N2 effect*) OR (side N2 effect*) OR (undesirable N2 effect*) OR (injurious N2 effect*) OR (patient N2 safety) OR (patient N2 harm) OR (medical N2 error*) OR unintended OR unexpected OR threat* OR (secondary N2 effect*)) OR (SU ((patient N2 risk*) OR (adverse N2 effect*) OR (side N2 effect*) OR (undesirable N2 effect*) OR (injurious N2 effect*) OR (patient N2 safety) OR (patient N2 harm) OR (medical N2 error*) OR unintended OR unexpected OR threat* OR (secondary N2 effect*)) OR MH quality improvement OR MH Quality of Health Care+ OR (TI (quality N2 improvement) OR “quality of care” OR “quality of health care” OR “quality of healthcare”) OR (AB (quality N2 improvement)) OR (SU (quality N2 improvement))) AND (MH Telehealth+ OR (TI (telemedicine OR Mobile N2 Health OR mHealth OR telehealth OR eHealth OR e-health OR e-medicine OR emedicine OR etherapy OR e-therapy OR (web-based N2 application*) OR (Web N2 based N2 app*) OR (web N2 based N2 self N2 help) OR (web N2 based N2 self N2 management) OR (web N2 based N2 self N2 monitoring) OR (web N2 based N2 self N2 report) OR (web N2 based N2 self N2 screening) OR (Web N2 based N2 Intervention*) OR (internet N2 delivered) OR (blended N2 care) OR telecare OR Telerehabilitation* OR (Virtual N2 Rehabilitation) OR (Virtual N2 Rehabilitations) OR Tele-rehabilitation OR (Tele N2 rehabilitation) OR Tele-rehabilitation* OR (Remote N2 Rehabilitation) OR (Remote N2 Rehabilitations) OR (Health N2 Information N2 Technology) OR (Health N2 Information N2 Technologies) OR (Digital N2 health) OR e-consult* OR econsult* OR (electronic N2 consult*) OR (remote N2 health*) OR (remote N2 consult*) OR (remote N2 counsel*) OR (remote N2 monitor*) OR (remote N2 therap*) OR (remote N2 treatment*) OR (medical N2 app) OR (medical N2 apps) OR (health N2 app) OR (health N2 apps) OR m-health OR (medical N2 health N2 technology) OR (Medical N2 application*) OR (health N2 application*) OR ((telemedicine OR Mobile Health OR mHealth OR telehealth OR eHealth OR e-health OR e-medicine OR emedicine OR etherapy OR e-therapy OR telecare OR Telerehabilitation*) AND (risk OR risks)))) OR (AB (telemedicine OR Mobile N2 Health OR mHealth OR telehealth OR eHealth OR e-health OR e-medicine OR emedicine OR etherapy OR e-therapy OR (web-based N2 application*) OR (Web N2 based N2 app*) OR (web N2 based N2 self N2 help) OR (web N2 based N2 self N2 management) OR (web N2 based N2 self N2 monitoring) OR (web N2 based N2 self N2 report) OR (web N2 based N2 self N2 screening) OR (Web N2 based N2 Intervention*) OR (internet N2 delivered) OR (blended N2 care) OR telecare OR Telerehabilitation* OR (Virtual N2

	Rehabilitation) OR (Virtual N2 Rehabilitations) OR Tele-rehabilitation OR (Tele N2 rehabilitation) OR Tele-rehabilitation* OR (Remote N2 Rehabilitation) OR (Remote N2 Rehabilitations) OR (Health N2 Information N2 Technology) OR (Health N2 Information N2 Technologies) OR (Digital N2 health) OR e-consult* OR econsult* OR (electronic N2 consult*) OR (remote N2 health*) OR (remote N2 consult*) OR (remote N2 counsel*) OR (remote N2 monitor*) OR (remote N2 therap*) OR (remote N2 treatment*) OR (medical N2 app) OR (medical N2 apps) OR (health N2 app) OR (health N2 apps) OR m-health OR (medical N2 health N2 technology) OR (Medical N2 application*) OR (health N2 application*))) OR (SU (telemedicine OR Mobile N2 Health OR mHealth OR telehealth OR eHealth OR e-health OR e-medicine OR emedicine OR etherapy OR e-therapy OR (web-based N2 application*) OR (Web N2 based N2 app*) OR (web N2 based N2 self N2 help) OR (web N2 based N2 self N2 management) OR (web N2 based N2 self N2 monitoring) OR (web N2 based N2 self N2 report) OR (web N2 based N2 self N2 screening) OR (Web N2 based N2 Intervention*) OR (internet N2 delivered) OR (blended N2 care) OR telecare OR Telerehabilitation* OR (Virtual N2 Rehabilitation) OR (Virtual N2 Rehabilitations) OR Tele-rehabilitation OR (Tele N2 rehabilitation) OR Tele-rehabilitation* OR (Remote N2 Rehabilitation) OR (Remote N2 Rehabilitations) OR (Health N2 Information N2 Technology) OR (Health N2 Information N2 Technologies) OR (Digital N2 health) OR e-consult* OR econsult* OR (electronic N2 consult*) OR (remote N2 health*) OR (remote N2 consult*) OR (remote N2 counsel*) OR (remote N2 monitor*) OR (remote N2 therap*) OR (remote N2 treatment*) OR (medical N2 app) OR (medical N2 apps) OR (health N2 app) OR (health N2 apps) OR m-health OR (medical N2 health N2 technology) OR (Medical N2 application*) OR (health N2 application*))))
