# Supplementary material for: eHealth Apps Replacing or Complementing Health Care Contacts: Scoping Review on Adverse Effects
Source: J Med Internet Res. 2019 Mar 1;21(3):e10736. doi: 10.2196/10736 (PMC6421717; doi:10.2196/10736)
Supplement: Multimedia Appendix 2 [file jmir_v21i3e10736_app2.pdf]

Study design	Author												
		**Was there a clear statement of the aim of the research**	**Is a qualitative methodology appropriate**	**Was the research design appropriate to address the aims of the research?**	**Was the recruitment strategy appropriate to the aims of the research?**	**Was the data collected in a way that addressed the research issue?**	**Has the relationship between researcher and participants been adequately considered?**	**Have ethical issues been taken into consideration?**	**Was the data analysis sufficiently rigorous?**	**Is there a clear statement of findings?**	**How valuable is the research?**		**Score Yes out of the total number of questions**
**Qualitative**	Benvenutti 2014 [24]	y	c	n	y	c	n	y	y	y	L		5/9
	Bodker 2015 [25]	y	y	n	n	n	n	y	n	y	P		4/9
	Cady 2015 [29]	y	c	y	n	y	n	n	y	y	L		5/9
	Chang 2013 [26]	y	y	y	c	y	n	y	n	n	L		5/9
	Duggan 2015 [31]	y	y	n	n	y	y	y	y	n	L		6/9
	Fairbrother 2013 [27]	y	y	y	y	y	y	y	y	y	H		9/9
	Fatehi 2015 [30]	y	y	c	c	y	n	y	n	y	L		5/9
	Mehrotra 2014 [32]	y	n	n	c	n	n	n	n	n	L		2/9
	Griffiths 2017 [34]	y	c	c	y	y	n	n	y	y	L		5/9
		**Did the trial address a clearly focused issue?**	**Was the assignment of patients to treatments randomised?**	**Were all of the patients who entered the trial properly accounted for at its conclusion?**	**Were patients, health workers and study personnel 'blind' to treatment?**	**Were the groups similar at the start of the trial?**	**Aside from the experimental intervention, were the groups treated equally?**	**How large was the treatment effect?**	**How precise was the estimate of the treatment effect?**	**Can the results be applied in your context? (or to the local population?)**	**Were all clinically important outcomes considered?**	**Are the benefits worth the harms and costs?**	
**RCT**	Buvik 2016 [28]	y	y	y	n	y	y	n/a	c	y	y	c	7/9
	Petrella 2014 [33]	y	y	y	n	y	y	n/a	c	y	y	y	8/9

Y= yes, N= no, C=Can't tell, n/a= not applicable.; L=Low, H= High, P= Pilot,
