# Supplementary material for: eHealth Apps Replacing or Complementing Health Care Contacts: Scoping Review on Adverse Effects
Source: J Med Internet Res. 2019 Mar 1;21(3):e10736. doi: 10.2196/10736 (PMC6421717; doi:10.2196/10736)
Supplement: Multimedia Appendix 3 [file jmir_v21i3e10736_app3.docx]

Description of the 11 included articles

|  | Author  Year Country | Title | Name eHealth  Intervention  and Function | Study Participants | Study design | Outcome concerning adverse effects and Measurements | Results  concerning adverse effects | Author’s conclusion  concerning adverse effects | CASP  Score^a^ |
| --- | --- | --- | --- | --- | --- | --- | --- | --- | --- |
|  |  |  |  |  |  |  |  |  |  |
| 1 | Benvenuti et al.  2014  Italy  [23] | Community-based exercise for upper limb paresis: a controlled trial with telerehabilitation | Telerehabiltatio:  Supporting physical Exercise | Patients with upper limb paresis secondary to stroke  n= 143 Intervention n= 45 Control | Effectiveness study with geographic control group | - Spontaneously reported or observed study-related adverse events   Observation by investigator or staff | - No study-related   adverse events | The intervention is safe | 5/9 |
| 2 | Bodker et al.  2015  Denmark  [24] | Providing rehabilitation online - invisible work and diagnostic agents | Telecare:  Supporting physical Exercise | Patients with very severe COPD, pulmonary function<30%  n= 4 patients  n= 1 nurse  n= 1 physical therapist | Qualitative  study | - Socio-technical workings - Emergence of invisible work - Negotiation of agency   Semi structured interviews | - A new perceptual distance between patients and therapists - New time-consuming work routines - Responsibilities less transparent | Telecare technologies cannot be expected simply to increase the efficiency and quality of healthcare services in uniform ways because several new work routines emerge and because of its complex implications for the actions played out in the telecare space | 4/9 |
| 3 | Buvik et al.  2016  Norway  [27] | Quality of care for remote orthopaedic consultatios  using telemedicine: a randomised controlled trial | Telemedicine consultation:  Consultation | Patients with orthopaedic problems  n= 199 Intervention n= 190 Control | RCT | - Reported adverse events - Consultation Duration - Re-referrals   Patient questionnaire  Providers questionnaire  (Non- validated) | - No serious adverse advents are found related to the mode of the consultation - Mean consultation duration not different - No difference in re-referrals | It was safe to offer video-assisted remote consultations | 7/9 |
| 4 | Cady et al.  2013  USA  [28] | Mixed Methods Approach for Measuring the impact of Video Telehealth on Outpatient Clinic Triage Nurse Workflow | Video Telehealth:  Triage | Children with complex conditions  n= 11 Families  n= 2 Registered nurses | Mixed methods protocol | - Nurse workflow - Interactions of nurses with persons and artifacts   Observation ,  semi-structured interviews,  documentary study  time motion study | Increased triage time by   - Increased depth breath and quality of data - Increased frequency of communication with clinician - Disruption by unpredictable connectivity issues | Less efficient because of increased workflow | 5/9 |
| 5 | Chang et al.  2013  Taiwan  [25] | Telecare for Diabetes Mellitus | Telecare:  Supporting self-management  Consultation | Patients with Diabetes  n= 9 nurses | Qualitative study | - Nurses concerns   Semi- structured in-depth interviews | - Poor cooperation of the patient - Sense of losing privacy - Stigmatization | Nurses must be cautious with confidentiality issues and potential negative feelings resulting from the changing nurse-patient relationship | 5/9 |
| 6 | Duggan et al.  2015  UK  [30] | Qualitative evaluation of the SMART2 self-management system for people in chronic pain | Internet intervention:  Supporting self-management | People in chronic pain  n= 8 patients | Qualitative study | - User’s negative comments   Semi-structured Interviews | - Loss of trust - Lack of willingness - Lack of human face to face contact | Complex programmes of therapeutic exercises delivered by technology had limited success in engaging people in chronic pain | 6/9 |
| 7 | Fairbrother et al.  2013  UK  [26] | Exploring tele monitoring and self-management by patients with chronic obstructive pulmonary disease: a qualitative study embedded in a randomized controlled trial | Telemonitoring: Supporting self-management | Patients with COPD  n= 38 patients,  n= 32 healthcare professionals | Qualitative study embedded within RCT | Patient and professional experiences   - Impact on selfcare - Dynamics of the patient-practitioner relationship   Semi-structured Interviews | - Concerns about promoting the sick role - Creates dependence on telemonitoring and professionals | Patients and professionals need to work together to define and delineate their roles and responsibilities in relation to telemonitoring supported self-management | 9/9 |
| 8 | Fatehi et al.  2015  Australia  [29] | Patient satisfaction with video teleconsultation in a virtual diabetes outreach clinic | Video Teleconsultation:Consultation | People with diabetes  n = 24 patients | Cross-sectional observational survey | - Patient satisfaction   Observational survey  Semi-structured and open ended questionnaire  (Non validated) | - Lack of physical contact causes concerns about long term complications of diabetes (21% of respondents) | The overall proportion of patients with concerns about the lack of physical contact was low. The endocrinologist can work in partnership with the general practitioner or a trained nurse when physical examination is needed. | 5/9 |
| 9 | Griffiths et al.  2017  UK  [33] | Timely Digital Patient-Clinician Communication in Specialist Clinical Services for Young People: A mixed-Methods Study( The LYNC Study) | Digital Communication:  Communication about clinical issues | Young people (16-24) with long-term conditions  n =165 patients  n =79 clinical team members  n =173 clinical team members  n=16 information specialists | Mixed-methods | - Patient safety   Interviews | - Communication failures - Failure to record the content of the communication - Failure to consult the patient’s notes prior to engaging in communication | Clinical teams need a proactive approach to ethics, governance, and patient safety when using digital communication | 5/9 |
| 10 | Mehrotra et al.  2014  USA  [31] | A comparison of care at e-visits and physician office visits for sinusitis and urinary tract infection | eVisits:  Care | Patients with Sinusitis  Patients with UTI^b^  n= 465 Sinusitis  n= 99 UTI | Retrospective research | - Ordering of tests - Follow- up visits - Antibiotic prescribing   Inspection of diagnosis codes | - Less likely to order UTI relevant tests - No difference for follow-up visits - Overprescribing of antibiotics - Less likely to order preventive care | When physicians cannot directly examine the patient, they may apply a “conservative” approach and order antibiotics | 2/9 |
| 11 | Petrella et al.  2014  Canada  [32] | Mobile health, exercise and metabolic risk: a randomized controlled trial | Mobile health (mHealth):  Supporting physical exercise | People with at least two metabolic syndrome risk factors  n= 67 Intervention n= 60 Control | RCT | - SBP^c^ at 12 weeks - SBP at 52 weeks   Systolic blood pressure measure | - Reduction SBP greater in control group at 12 weeks and similar at 52 weeks - All secondary outcomes Similair between groups | In participants with increased cardiometabolic risk, exercise prescription alone had greater short-term improvements in systolic blood pressure compared to the mHealth intervention, though over 52 weeks improvements were equal | 8/9 |

- ^a^Study quality: we used Critical Appraisal Skills Programme (2017) checklist i.e. Qualitative Research ( CASP qual)and checklist RCT (qual RCT) score indicated by xxx out of ten questions answered with yes .
- ^b^ UTI: Urinary Tract Infections
- ^c^ SBP: systolic blood pressure
